# Data for 3D printing enlarged museum specimens for the visually impaired

**DOI:** 10.46471/gigabyte.3

**Published:** 2020-07-01

**Authors:** Anton du Plessis, Johan Els, Stephan le Roux, Muofhe Tshibalanganda, Toni Pretorius

**Affiliations:** ^1^ CT Scanner Facility, Stellenbosch University, Stellenbosch, South Africa 7602; ^2^ Department of Mechanical Engineering, Central University of Technology, Bloemfontein, South Africa 6001; ^3^ Bruker microCT, Kontich, Belgium; ^4^ National Museum, Bloemfontein, South Africa 9300

## Abstract

Museums are embracing new technologies and one of these is the use of 3D printing. 3D printing allows for creating physical replicas of items which may, due to great value or significance, not be handled by the public, or which are too small or fragile to be handled or even seen with the naked eye. One such application of new technologies has been welcomed by the National Museum in Bloemfontein, Free State, South Africa. Here, blown-up (enlarged) Museum specimens were 3D printed for various interactive exhibits that are aimed at increasing the accessibility of their permanent displays for visually impaired visitors who rely greatly on touch as a source of observation. A selection of scorpions, pseudoscorpions, mites and archetypal bird skulls were scanned, processed and 3D printed to produce enlarged, highly functional nylon models. This data paper provides the raw micro Computed Tomography (micro-CT) scan data and print ready STL files processed from this data. The STL files may be used in their current format and details of the printing are provided.

## Introduction

In general the use of 3D printed models for museum displays or research has been positively received as evidenced by numerous publications and in some instances, a preference for 3D printed models was indicated [1, 2]. The use of micro Computed Tomography (micro-CT) in biological sciences was reviewed in [3] which also includes data sets of a Jackson’s chameleon with 3D print files [4]. The use of micro-CT and 3D printing has been used successfully for non-invasive investigations as in [5, 6] where 3D print replicas of the contents of an Egyptian mummified falcon were reproduced successfully.

## Materials and Methods

MicroCT and nanoCT scans were conducted at the Stellenbosch CT facility [7]. The smallest samples were scanned in the nanoCT and samples > 10 mm wide were scanned in the microCT instruments using optimized settings and voxel sizes appropriate to each sample. Image data was analyzed in Volume Graphics VGSTUDIO MAX 3.2 (VG Studio MAX, RRID:SCR_017997). A de-noising was applied, followed by a surface determination function. In cases of noisy data, some cleanup was done according to visual inspection using morphological image tools (erode/dilate and region growing). The final STL (stereolithography) files were then processed in Materialise Magics (23.0.1.19 64 bit) software to remove loose shells and reduce the number of triangles to ensure clean 3D models that can easily be scaled to any specific requirement.

The clean 3D models were produced in an EOS P385 Selective Laser Sintering system in white PA2200 Polyamide (Nylon) powder.

## Data

**Figure 1.**
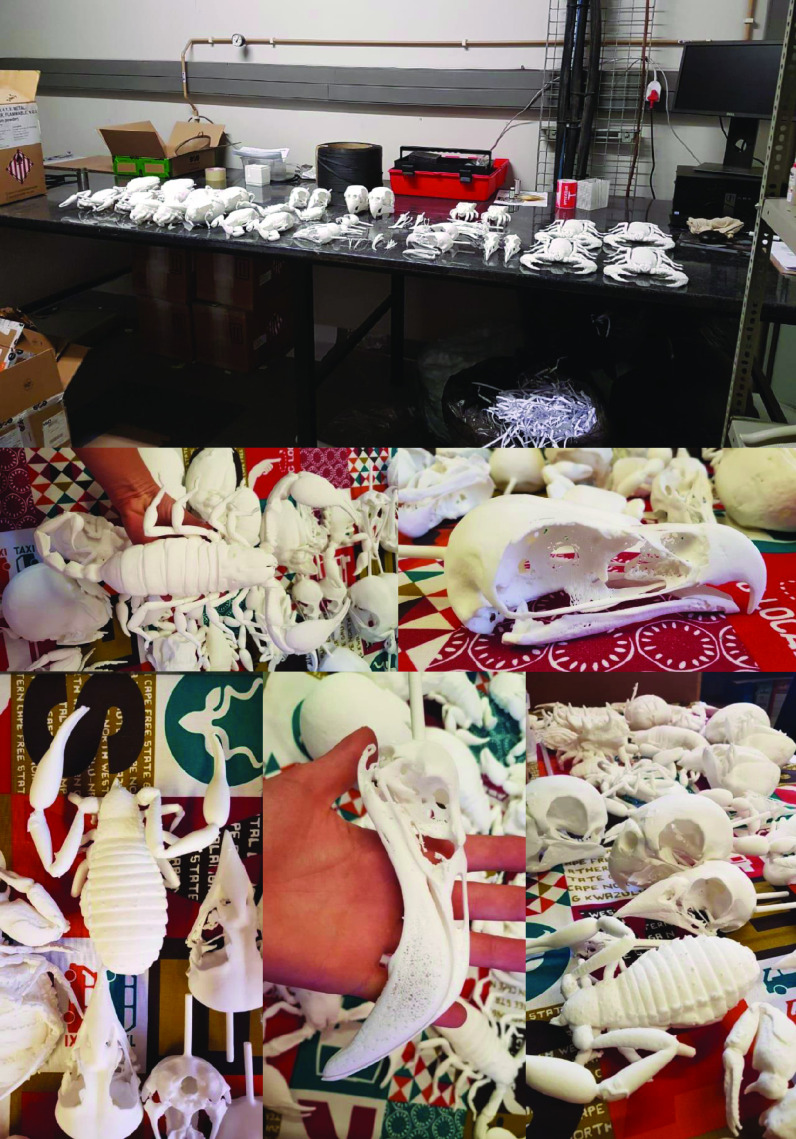
Photos of the final printed models for the Museum display, ready for surface finishing.

**Figure 2.**
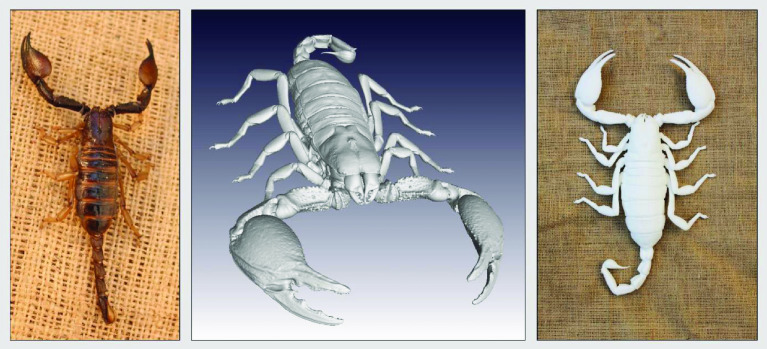
*Opistophthalmus carinatus* (Robust burrower) production process. From left to right: Wet specimen (approximately 100 mm), STL image, Nylon print (350 mm).

Photos of the final 3D printed objects are shown in Figure 1 with a comparative visual of a larger scorpion specimen in the three stages of the replication process found in Figure 2. The CT data is provided in the form of image stacks with associated voxel size and other scan settings in the accompanying text file.

**Figure 3.**
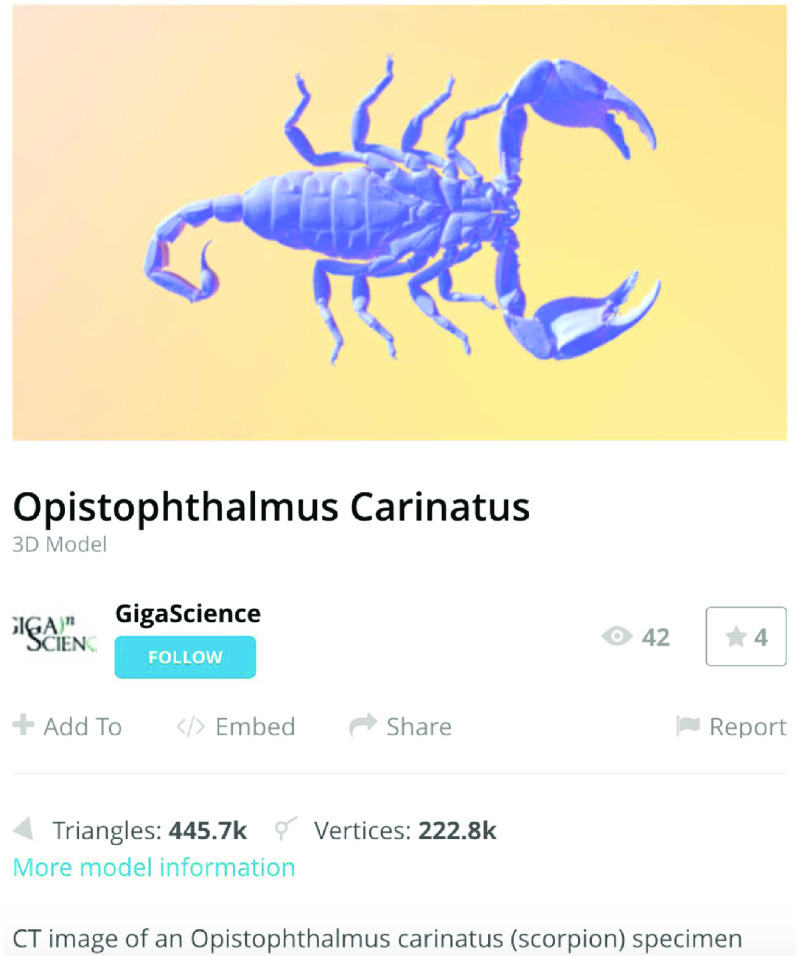
Interactive CT image of an Opistophthalmus carinatus (scorpion) specimen in sketchfab https://sketchfab.com/3d-models/opistophthalmus-carinatus-4b99189013b1478aadf43b195c522e17

The processed STL files used for printing are also provided with each scan data set and can be viewed with any 3D model viewer The summary of all data provided is given in Table 1. Interactive views of the 3D models are available in the sketchfab repository, which enables CT images to be interactively explored. Figure 3 provides an example of an interactive CT sketchfab view of the *Opistophthalmus carinatus* specimen.

**Table 1:** Data description summary, image stacks all 16 bit.

**Scientific name (Taxon ID)**	**Image**	**Voxel size**	**Image stack size**	**STL file size**
**Arachnida**				
**Scorpiones**				
*Opistophthalmus carinatus* (NCBI: txid190115)	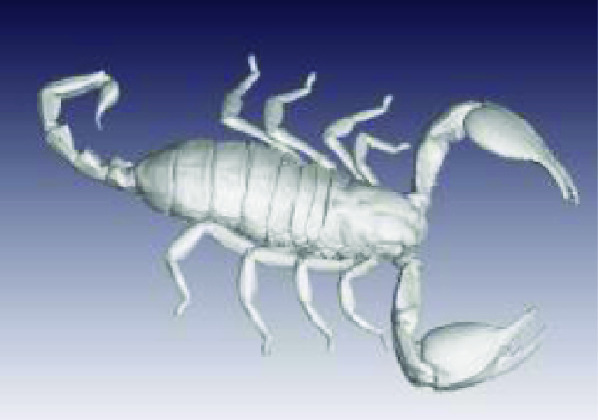	0.08000000	960 MB (1,007,004,206 bytes)	21 763 KB
**Pseudoscorpiones**				
Cheliferidae: *Beierius walliskewi* (GBIF ID: 2126726)	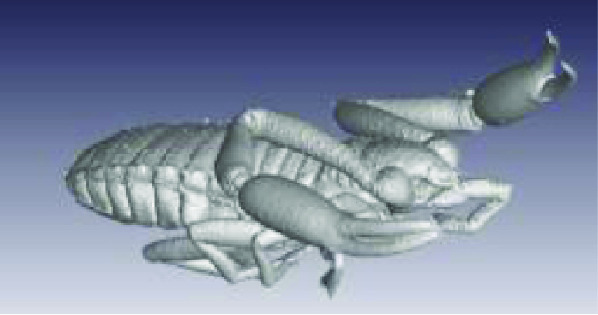	0.00399991	1.93 GB (2,080,320,334 bytes)	15 788 KB
Atemnidae: *Titanatemnus natalensis* (GBIF ID: 2126451)	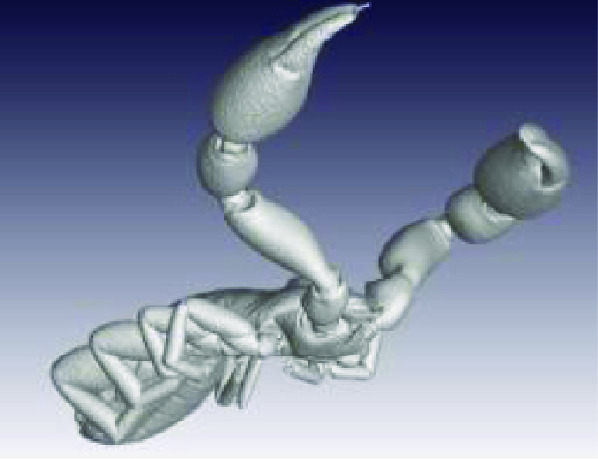	0.00700001	2.31 GB (2,484,388,396 bytes)	12 272 KB
Feaeallidae: *Feaella capensis* (NCBI: txid2590491)	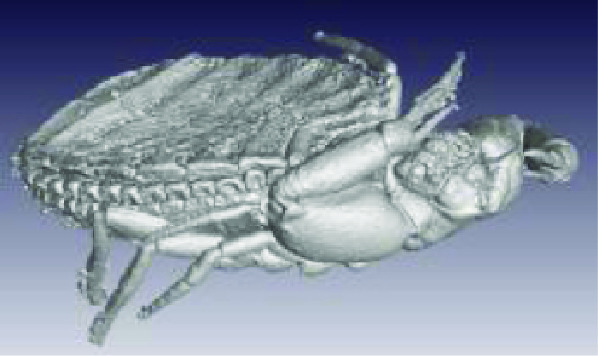	0.00199991	2.99 GB (3,218,034,562 bytes)	91 239 KB
Olpiidae: *Horus obscurus* (GBIF ID: 2125067)	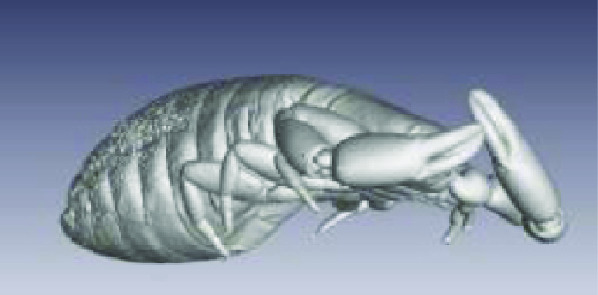	0.00399991	2.22 GB (2,394,603,554 bytes)	14 279 KB
**Acari**				
Oribotritiidae: *Indotritia retusa* (GBIF ID: 2188784)	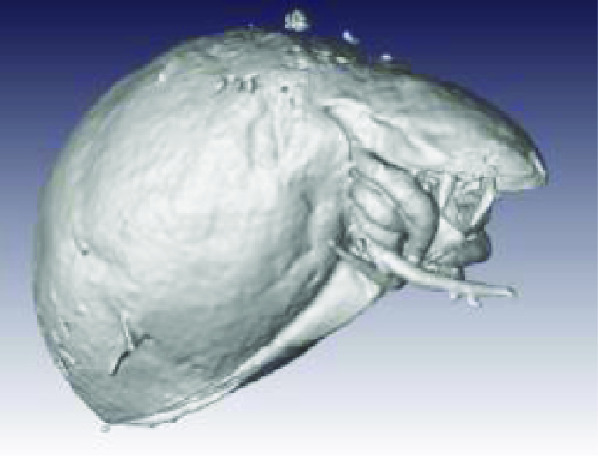	0.00200000	387 MB (405,960,582 bytes)	15 131 KB
Nothridae: *Nothrus anauniensis* (NCBI: txid1685385)	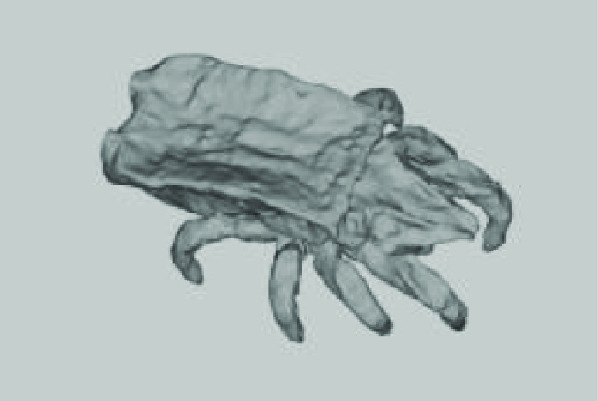	0.00199991	499 MB (524,264,126 bytes)	8 263 KB
Neoliodidae: *Neoliodes terrestris* (GBIF ID: 2192854)	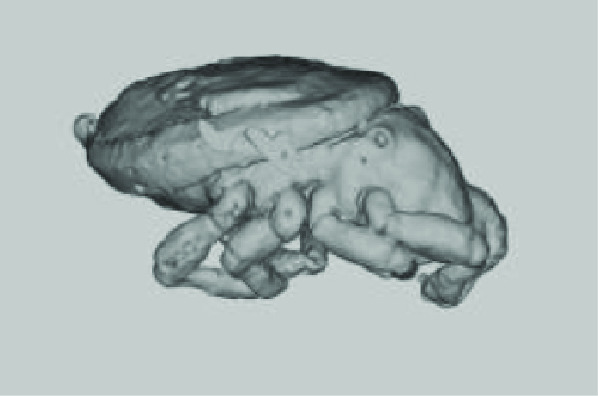	0.00199991	1.06 GB (1,144,736,558 bytes)	10 138 KB
Galumnidae: *Galumna capensis* (GBIF ID: 2193431)	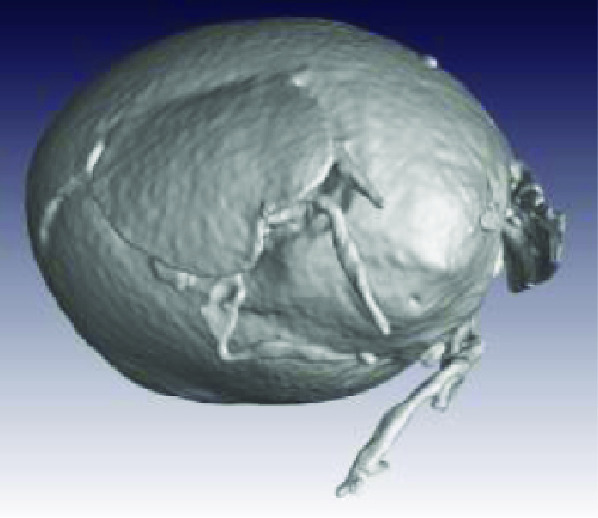	0.00199991	760 MB (797,371,236 bytes)	5 721 KB
Crotoniidae: *camisia hamulifera* (GBIF ID: 2192175)	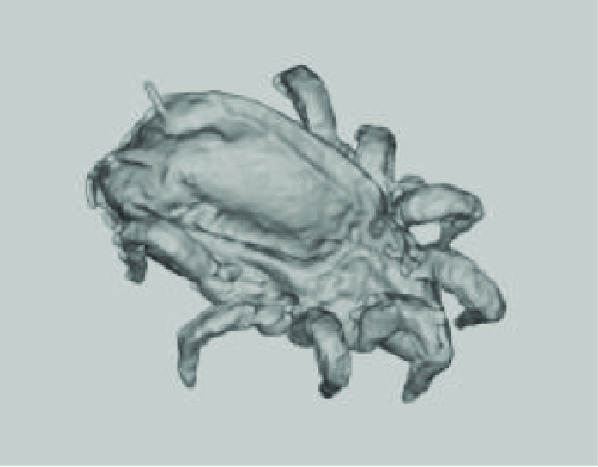	0.00199991	1.13 GB (1,222,213,194 bytes)	12 717 KB
**Aves**				
Accipitridae: *Aquila verreauxii* (NCBI: txid252782)	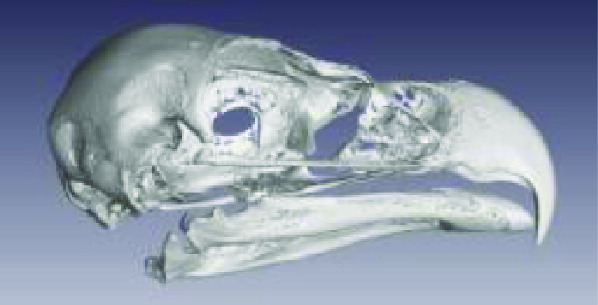	0.07999998	2.20 GB (2,365,352,542 bytes	54 682 KB
Ploceidae: *Euplectes progne* (NCBI: txid221973)	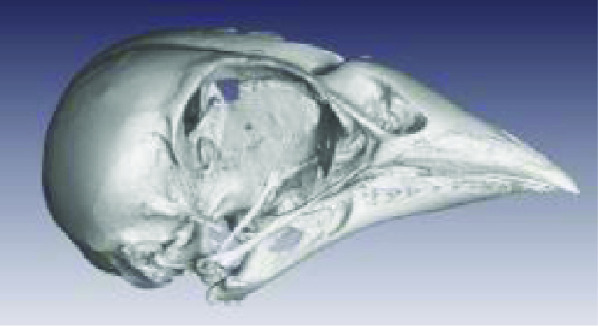	0.02700000	2.25 GB (2,427,044,488 bytes)	150 389 KB
Psittacidae: *Agapornis roseicollis* (NCBI: txid60468)	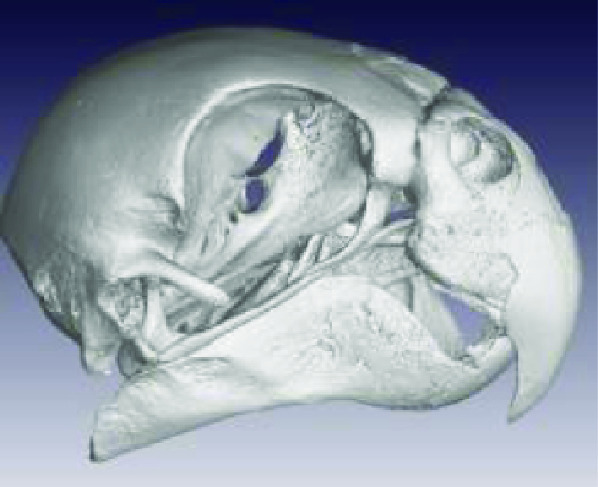	0.03300000	2.62 GB (2,822,618,674 bytes)	208 756 KB
Hirundinidae: *Hirundo spilodera* (NCBI: txid317141)	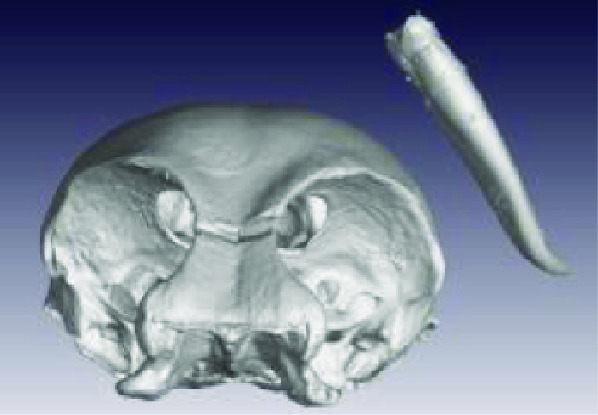	0.02000000	2.86 GB (3,079,714,774 bytes)	n/a
Sturnidae: *Onychognathus morio* (NCBI: txid381114)	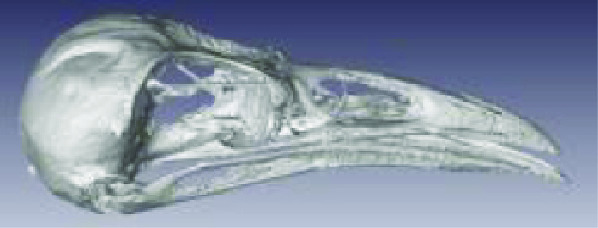	0.04000000	1.61 GB (1,738,054,024 bytes)	81 143 KB
Threskiornthidae: *Plegadis falcinellus* (NCBI: txid52788)	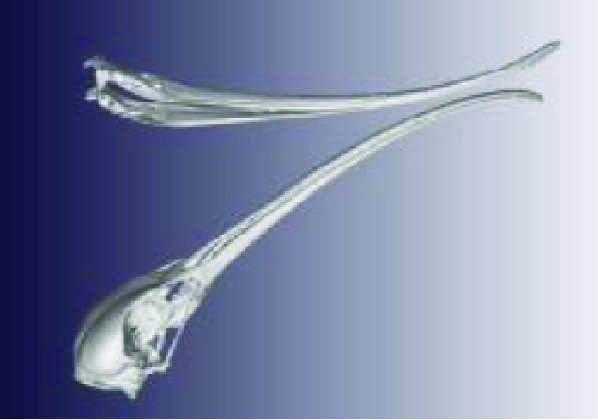	0.09833335	2.01 GB (2,160,723,314 bytes)	3 520 KB
Threskiornthidae: Platalea *alba* (NCBI: txid33578)	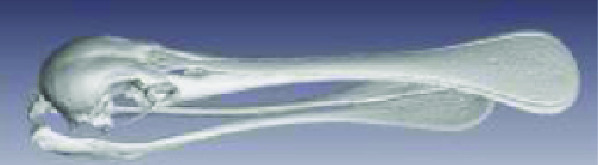	0.15014335	681 MB (714,566,426 bytes)	81 073 KB
Laridae: *Larus californicus* (NCBI: txid126681)	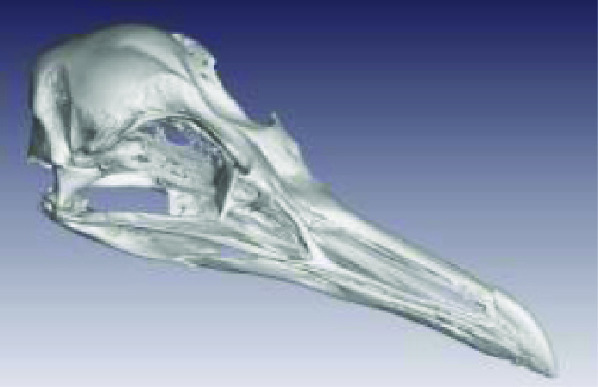	0.06999998	1.33 GB (1,436,767,642 bytes)	39 469 KB
Phoenicopteridae: *Phoenicopterus ruber* (NCBI: txid9217)	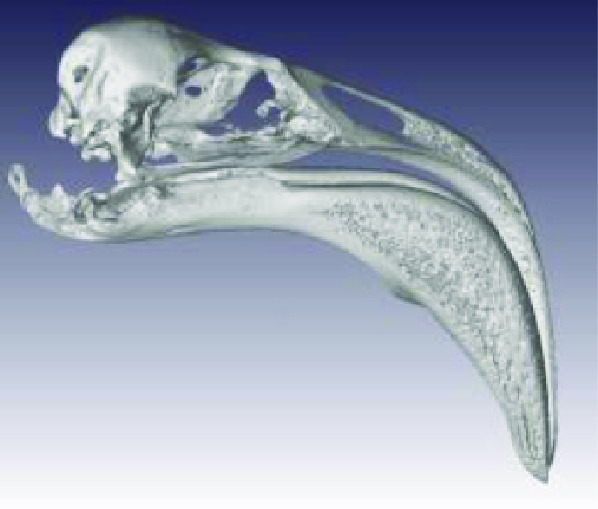	0.09800000	3.24 GB (3,485,882,170 bytes)	71 855 KB
Promeropidae: *Promerops gurneyi* (NCBI: txid670928)	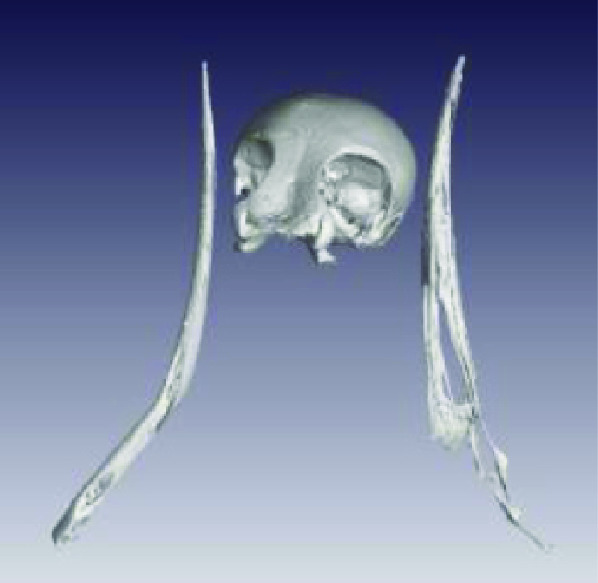	0.03333333	4.64 GB (4,991,580,140 bytes)	57 879 KB

**Figure 4.**
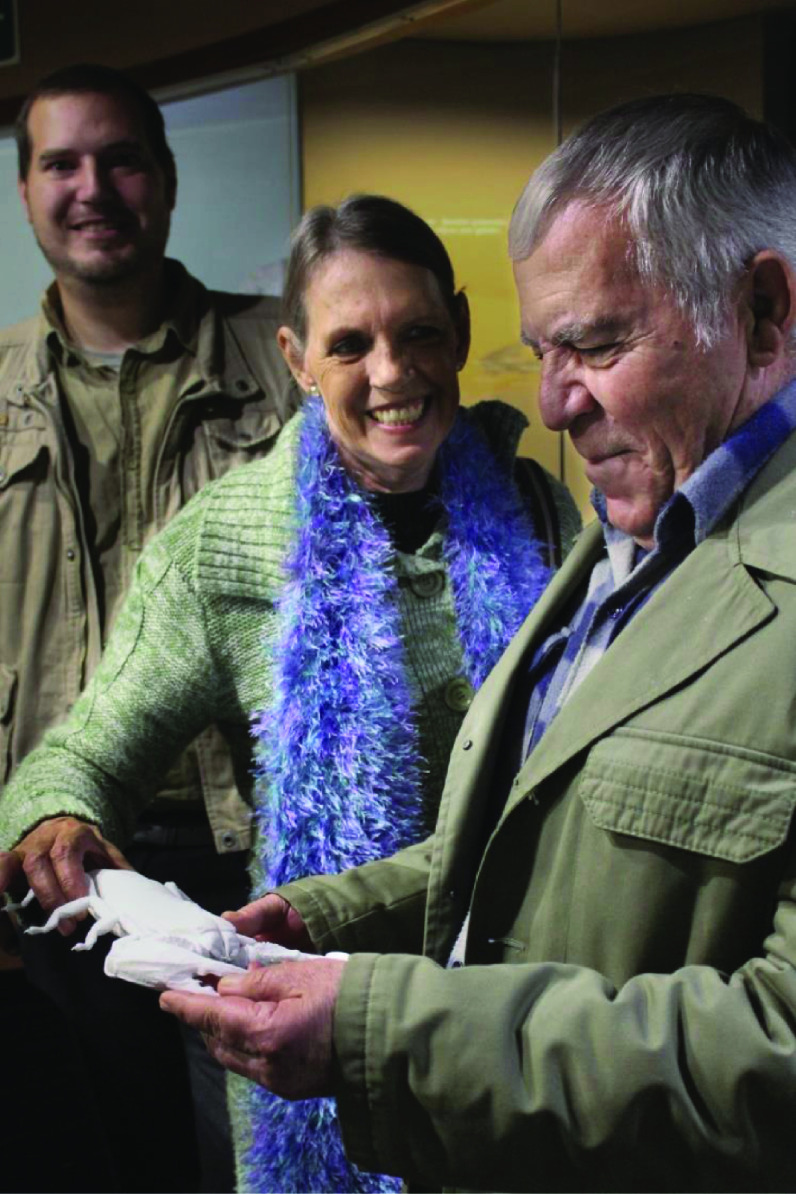
Members of the Free State Society for the Blind putting the 3D models through a trial run at a recent visit to the Museum. From left to right: Jan Andries Neethling (Museum Arachnologist), Anne de Beer, Pannie de Beer (Rendezvous Support Group).

Creating these 3D models enabled the National Museum in Bloemfontein to create a new type of exhibit, where visually-impaired visitors can handle the 3D printed replicas to better understand the specimens that previously they could only learn about through verbal or written descriptions (Figure 4). Creating and having these resources increases the accessibility of the museum’s information to a broader community than was previously served.

## Conclusions

This data will be useful for Museums setting up similar display. In addition to museum activities, these data can also be used for other research purposes that require material that cannot transported or shared. The workflow may also be useful for these and similar projects, and we urge others having similar types of data to make it openly available as well.

## Data Availability

All the data including CT scan details, 3D surface rendered images in STL format, and interactive views of the 3D models are available in the associated GigaDB dataset [8]. 3D models are also available for view in Sketchfab (https://sketchfab.com/GigaDB/collections/3d-printing-data-from-enlarged-museum-specimens), and for download by the 3D printing community in thingiverse (https://www.thingiverse.com/thing:3869332).

## Abbreviations

GB: gigabytes; KB: kilobytes, MB: megabytes; micro-CT: micro Computed Tomography; STL: stereolithography files
